# Using qualitative methods to inform the trade-off between content validity and consistency in utility assessment: the example of type 2 diabetes and Alzheimer's Disease

**DOI:** 10.1186/1477-7525-8-23

**Published:** 2010-02-12

**Authors:** Clare Mcgrath, Diana Rofail, Elizabeth Gargon, Linda Abetz

**Affiliations:** 1 Health Technology Assessment Policy, Medical Division, Worldwide Pharmaceutical Operations, 3-1-60 Walton Oaks, Tadworth, Surrey KT20 7NS, UK; 2 Mapi Values, Adelphi Mill, Bollington, Macclesfield, Cheshire, SK10 5JB, UK

## Abstract

**Background:**

Key stakeholders regard generic utility instruments as suitable tools to inform health technology assessment decision-making regarding allocation of resources across competing interventions. These instruments require a 'descriptor', a 'valuation' and a 'perspective' of the economic evaluation. There are various approaches that can be taken for each of these, offering a potential lack of consistency between instruments (a basic requirement for comparisons across diseases). The 'reference method' has been proposed as a way to address the limitations of the Quality-Adjusted Life Year (QALY). However, the degree to which generic measures can assess patients' specific experiences with their disease would remain unresolved. This has been neglected in the discussions on methods development and its impact on the QALY values obtained and resulting cost per QALY estimate underestimated. This study explored the content of utility instruments relevant to type 2 diabetes and Alzheimer's disease (AD) as examples, and the role of qualitative research in informing the trade-off between content coverage and consistency.

**Method:**

A literature review was performed to identify qualitative and quantitative studies regarding patients' experiences with type 2 diabetes or AD, and associated treatments. Conceptual models for each indication were developed. Generic- and disease-specific instruments were mapped to the conceptual models.

**Results:**

Findings showed that published descriptions of relevant concepts important to patients with type 2 diabetes or AD are available for consideration in deciding on the most comprehensive approach to utility assessment. While the 15-dimensional health related quality of life measure (15D) seemed the most comprehensive measure for both diseases, the Health Utilities Index 3 (HUI 3) seemed to have the least coverage for type 2 diabetes and the EuroQol-5 Dimensions (EQ-5D) for AD. Furthermore, some of the utility instruments contained items that could not be mapped onto either of the proposed conceptual models.

**Conclusions:**

Content of the utility measure has a significant impact on the treatment effects that can be observed. This varies from one disease to the next and as such contributes to lack of consistency in observable utility effects and incremental utility scores. This observation appears to have been omitted from the method development considerations such as reference methods. As a result, we recommend that patients' perspectives obtained via qualitative methods are taken into consideration in the ongoing methods development in health state descriptions for generic utility instruments. Also, as a more immediate contribution to improving decision making, we propose that a content map of the chosen utility measure with patient-reported domains be provided as standard reporting in utility measurement in order to improve the transparency of the trade-offs in relation to patient relevance and consistency.

## Background

National, regional and local decision makers have to determine how best to allocate scarce resource so as to obtain optimum benefit [1]. The Quality-Adjusted Life Year (QALY) is a concept used to facilitate decision making in that it is intended to allow comparison of health effects across different diseases [2].

In the United Kingdom, The National Institute for Health and Clinical Excellence (NICE) guidance clearly states that for cost effectiveness analysis, the value of health effects should be expressed in terms of QALYs and the measurement of changes in health-related quality of life (HRQoL) should be reported directly from patients. Although the NICE guidelines state the use of disease-specific preference based measures may be considered if they are justified, the value of changes in patients' HRQoL (that is, utilities) should be based on public preferences using a choice-based method [3].

The equivalent body for Scotland, Scottish Medicine Consortium (SMC), also prefer the QALY and consider it to be the most appropriate generic assessment of health benefit that reflects both mortality and HRQoL effects, and which allows comparisons between interventions [4]. Likewise, reimbursement bodies in Sweden, the Netherlands and Canada all clearly specify QALYs as the preferred method [5-7].

QALYs are calculated by estimating the total life years gained from a treatment and then weighting each year with a score. This score ranges from 0 to 1 (or 100) representing 'worst imaginable health' and 'best health' respectively. The utility is considered reflective of HRQoL in that year. The fundamental idea is that an extra year in good health does not have the same value to patients as a year in poor health [8]. The number of QALYs are then expressed as the value given to a particular health state multiplied by the period of time spent in that state to determine a composite measure of health [9].

This process requires a descriptor, a valuation, and a perspective for the utility measurement. There are various approaches that can be taken for each of these. For example, a descriptor method is a description of health and its impact on HRQoL and can be developed using clinicians, patients, or people from the general population (or even a combination of these). The descriptor method can also be presented in different ways such as using a vignette or the EuroQol-5 Dimensions (EQ-5D) items. A valuation method refers to a value given to a health state description such as using a visual analogue scale or standard gamble, and a perspective can range from for example the healthcare provider or a societal perspective. The diverse approaches for descriptors, valuations and perspectives may contribute to a lack of consistency and diversity in scores, making consistent decision making more difficult.

Utility instruments incorporate preferences or values attached to individual health states and express health states as a single index. They can be classified as generic and therefore suitable for use in various populations and disease-specific populations, allowing decision makers to make comparisons. Examples of generic utility measures include the EQ-5D, the Health Utilities Index (HUI) [10], the 15-dimensional health related quality of life measure (15-D) [11] and the Medical Outcomes Study Health Survey (MOS SF-6D) [12]. The NICE guidelines specify that the (EQ-5D) is the preferred measure of utilities in adults [13].

Disease-specific instruments are developed to measure the patients' perceptions and HRQoL impact of a specific disease or health problem. The main advantages of disease-specific measures are that they tend to be relevant to the impact of a specific disease on patients, and clinicians find them useful [14]. In addition, in longitudinal studies, disease-specific instruments are more likely to be responsive to clinical changes since the issues assessed are so relevant to the condition [15]. However, the very nature of the measures being disease specific limits the ability to compare values across disease areas. Disease-specific utility instruments do not contain any items or health dimensions that are not relevant to the disease; these instruments have clear relevance to patients with the presenting problem and thus acceptability is likely to be high. Regulatory bodies such as Food and Drug Administration (FDA) and European Medicines Agency (EMA) have stated preferences for disease-specific measures which can show sensitivity in specific disease areas due to their relevance to patients and those who treat them; currently generic measures do not have this level of sensitivity for individual diseases. Thus, there is some degree of disconnect between regulatory bodies (such as FDA and EMA) and health technology bodies such as NICE or SMC. Opportunities to harmonize or integrate these methods could provide positive steps in reconciling and interpreting the different needs of decision makers.

Regulatory authorities such as the FDA and EMA have endorsed patient-reported outcomes and emphasised the need to take the patient perspective into account when developing an instrument, particularly aspects of HRQoL [16,17].

The recent special issue of the Value in Health summarising the ISPOR Consensus Development Workshop focuses on "moving the QALY forward" with emphasis on "how to define and refine the QALY" as a standard metric [18]. It is generally recognised that the sensitivity of the QALY can vary across different disease areas [4], that different methods of utility measurement yield different results and there is no clear consensus as to which instrument is the preferred method for utility elicitation [19]; although NICE have opted for the EQ-5D as a preferred instrument.

It was recently highlighted at the QALY development workshop that the absence of a better alternative makes the QALY an indispensable tool [20]. These generic instruments currently have a pivotal role in healthcare technology decision making in some countries and the outcome of such decisions that directly influence patients and carers.

One possible solution to developing a preferred approach is a 'reference method' [21] where a standardised approach (to descriptor, valuation method) would be proposed for all economic evaluations; while simultaneously not excluding other approaches. Although this is a theoretically appropriate approach, it is not yet clear how a reference method would be derived. It is also acknowledged that even if this was achieved, if a single measure was chosen, it could bias the allocation of resources in favour of some diseases or interventions that have impact on the particular attributes of that measure. This could be reduced by proposing an approach that included more than one measure but this was identified as being more costly. Qualitative methods could be helpful in deciding what should be encompassed in a "reference descriptor" as well as understanding the trade-off between content and ability to compare across diseases of the current utility descriptors.

The broad objective of this study was to assess how qualitative methods can improve knowledge about which current utility instruments assess concepts of relevance from the patients' perspective. Alzheimer's disease and type 2 diabetes were used as examples of diseases that are prevalent and represented in the general population.

## Methods

A literature review was performed in two parts. The aim of part one was to identify patient qualitative research in the two diseases of interest (type 2 diabetes and AD). The focus was to gain an understanding of the symptoms and subjective experiences of patients with type 2 diabetes and those with AD. Part two aimed to identify generic and disease-specific utility instruments used in type 2 diabetes and AD. The analysis then compared the relevant domains for the diseases with the construction and validation of the instruments.

### Literature Review Part I: Qualitative Research

A search strategy was implemented using electronic databases (PUBMED, PsycINFO, and Embase) to identify relevant qualitative studies from January 2003 to October 2008, using the search terms presented in Table 1. The review was limited to English language studies and human subjects.

**Table 1:** Search Terms

Terms	Keywords	Command
1. Indication*	Alzheimer's Disease [MeSH Terms] **OR **Diabetes mellitus, type 2 [MeSH Terms]	**AND**

2. Patient Reported Outcome (PRO)	Health-Related Quality of Life **OR **HRQoL **OR **hrql**OR **quality of life [MeSH Terms] **OR **QoL **OR **signs and symptoms [MeSH Terms] **OR **emotions [MeSH Terms] **OR **physical **OR **psychological **OR **psychosocial **OR **impact **OR **relationships **OR **family impact **OR **work [MeSH Terms] **OR **productivity **OR **absenteeism [MeSH Terms] **OR **presenteeism	**AND**

3. Method	Qualitative research [MeSH Terms] **OR **interviews as topic [MeSH Terms] **OR **narration [MeSH Terms]**OR **interview, psychological [MeSH Terms] **OR **grounded theory **OR **interpretive phenomenological analysis **OR **focus groups	

* Searches for each indication were performed independently.

On completion of the search all titles and abstracts were screened for possible inclusion in the study by two independent researchers. Disagreements were resolved by subsequent discussion with another researcher who performed an independent review and made a final decision.

To satisfy the inclusion criteria, selected abstracts had to include an appropriate clinical term related to one of the relevant indications (e.g. Diabetes mellitus, type 2 [MeSH Terms]), at least one patient-reported outcome term (e.g. quality of life), and at least one methodological term (e.g. qualitative research [MeSH Terms]) in the title or abstract.

Abstracts were excluded if they were primarily clinical, economic or quantitative in focus, the perspective was not patient focused (e.g. caregiver or health-care professional), or the abstract did not refer to a journal article (e.g. letters, conferences, dissertations, books or chapters).

Following pre-testing, data extraction tables were finalised to accurately extract information on the symptoms and subjective experiences of patients including patient quotes where relevant. A thematic analysis was performed on the patient quotes [22]. Each data item within each quote was examined repeatedly and was given equal attention during the coding process, which was thorough, inclusive and as comprehensive as possible. The coding process was performed without trying to fit it into a pre-existing coding frame. Rather, the relevant extracts from the dataset were collated to form themes. Themes were then checked against each other and against the original dataset to ensure that they were coherent, consistent, and distinctive. The process was predominantly inductive in that the themes identified were strongly linked to the data themselves, and thus data driven. Also, the themes were semantic themes identified within the explicit or surface meanings of the data, and the researcher was not looking for anything beyond what a patient said. However, there was gradual progression from description whereby the data were organised and summarised to show patterns, to an interpretative process to attempt to theorise on the significance of the patterns and their broader meanings and implications. The extracts were also checked to ensure that they matched the analytic claims. Following thematic analyses, conceptual models were developed for each indication to provide a visual representation of the key themes and how they appear to be related to each other.

### Literature Review Part II: Utility Measures

To identify utility studies, an additional search strategy was implemented from August 2006 to October 2008 using PUBMED, utilising the same indications as in Table 1, and a search for the following terms: utilities **OR **utility **OR **QALY [MeSH Terms]. To satisfy the inclusion criteria, papers had to include an appropriate clinical term and reference to a utility measure in the title or abstract. Limits and exclusions were similar to part one of the literature review. The extent to which patients and healthy people were involved in their development was considered. The utility instruments were then assessed for their relevance to the diabetes and AD populations.

### Mapping Utility Instruments to Proposed Conceptual Models

Each item (question) of utility instruments identified from part two of the literature review was then mapped on to the conceptual models. Each item was mapped where it was considered to reflect the concept, meaning that items could be mapped to more than one concept if deemed appropriate. Partial coverage was also considered, and items that partly covered concepts were mapped and indicated in brackets.

## Results

### Literature Review Part 1: Qualitative Research

**Figure 1:**
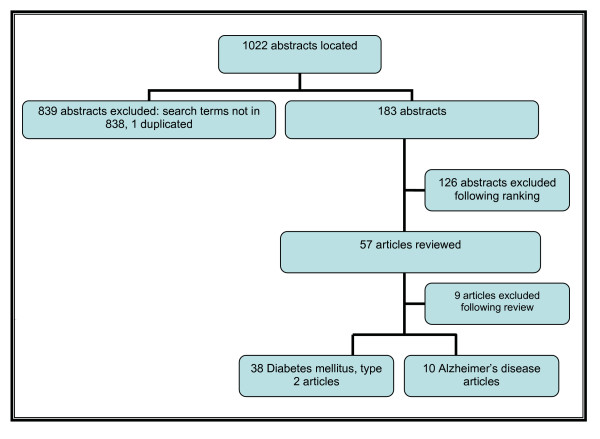
**Inclusion and Exclusion of Studies**.

The study selection process for part one of the literature review is presented in Figure 1.

### Patients Subjective Experiences with Type 2 Diabetes

Review of these studies revealed that aches and pains [23-25], hunger [23,26], thirst [23,26], tiredness [23,26,27], lack of energy [23,28,29], as well as dizziness [24,26] are prominent symptoms for patients with type 2 diabetes. Type 2 diabetes and associated symptoms were perceived to influence HRQoL in relation to physical functioning [23,30-33], role functioning [26,29,30,34-38], activities of daily living [23,27,32,39,40], mental health and mood [24,27,29,30,36,39,41-45], emotional functioning [23,25-27,30,31,34,36-38,40,41,44,46-54], relationships [27,31,32,41,49,55,56], sexual functioning [42,48,55], social functioning [27,31,32,37,41,42] and self image [23,29,31,33,42,44,47,51,53,57,58]. Table 2 provides example quotes to illustrate each of these concepts.

**Table 2:** Example quotes for type 2 diabetes concepts

Concept	Sub concept	Example quote
Symptoms	Tiredness	This morning I had a hard time just getting out of bed. I was so tired. Very very tired [27]
	
	Sweaty	Your body talks back to you...it reminds me. A lot of time I feel the diabetes is when it's low. I get sweaty, hot and hungry [23].
	
	Thirst	I used to feel thirsty at night time, but I used to think it was probably...because I had a very bad heating system..."[26]
	
	Hot	Your body talks back to you...it reminds me. A lot of time I feel the diabetes is when it's low. I get sweaty, hot and hungry [23].
	
	Hunger	Your body talks back to you...it reminds me. A lot of time I feel the diabetes is when it's low. I get sweaty, hot and hungry [23].
	
	Aches and pains	That's another meaning of diabetes - getting headaches, dizziness and body aches[24]
	
	Lack of energy	In the morning my energy runs out because my sugar is too low; in the evening it runs out because it is too high[23]
	
	Dizziness	I think it was like feeling dizzy, using the bathroom a lot, tired [26].

Emotional Functioning	Embarrassment	I am ashamed to do it [insulin therapy]. What will others think - that I am a drug addict or something?! [51]
	
	Frustration	I also have two small children and that's a problem...they don't like eatin' the way I eat...I get frustrated..." [34].
	
	Anger	It's the diabetic anger and people will not understand it...it's out of proportion with the event." [27]

Mental health	Anxiety	There are times when I'm here at home alone and I get so anxious as I start to think, 'I don't want to think about eating,' but it's the only thing on my mind - eating, eating, eating....and I start to cry because what's inside I know I cant eat [27].
	
	Depression	Sometimes, knowing that I am diabetic and that I am limited, sometimes if I'm not careful, it can cause depression[56]

Relationships	Partner	My husband does not want to pay attention and refuses to eat less salt and more vegetables and says the diet is disgusting[55]
	
	Friends	All of my friends, every time I run into one of them, the first thing they ask me is 'how's your diabetes?' Here I am trying to forget about my diabetes for a little while, and they remind me of it constantly [27].
	
	Family	Taking care of the kids. I want to have energy for them. You know I take them to the park and they want to play and I don't have enough energy to get up and play with them... [30]

Sexual Functioning	Activity	You get your tired periods, but...you just rest and then you're okay again. I don't have sex as much 'cos I'm tired more. [42]
	
	Desire	My sexual appetite was diminished. [42]
	
	Mental arousal	You're not turned on the same...you yearn for the feelings...but they're just not there[42]

Social Impact	Vacation/holidays	I cant do half of the things I used to...you cant go on vacations with people like you used to because you cant keep up with them...so I stay at home a lot. So then the depression - the whole circle starts again. [31]
	
	Socialising	It wasn't like that before diabetes. No I had a very active social life. I used to go to parties, I went out a lot - all the things someone normally does in his life. Now, I'm always tired. [27]
	
	Constraints	It puts time constraints on us...you're not just free to go out and have a day out to yourself. [31]

Role Functioning	Work	I want to go to work and I don't think I can manage a whole day of work. [30]
	
		Yeah, I guess I was tired. In fact I had a second job I had to stop doping it I was so tired. [26]

Physical Functioning	Activities	It makes me feel old, wasted. I use to go dancing every weekend. I wouldn't stop dancing. If I go to the dance hall, I would dance all the time and now, I cannot[30]
	
Self Image	Self concept	I am ashamed to do it. What will the others think - that I am a drug addict or something?! [51]
	
		I felt a bit like Frankenstein. I'm injecting someone here! You start looking at yourself more in the mirror and thinking Am I changing? [53].

Activities of Daily Living	Self care	You're always spilling urine, and that is too embarrassing. And you're never clean[23]
	
	Shopping	And you're never clean. That's why you can't go shopping...You know how to try on clothes[23]

### Patients Subjective Experiences with Alzheimer's Disease

Symptoms experienced by patients were mainly related to short term memory loss [59-63] and included loss of words and thought. In contrast to this there was a sense of physical well being [60,61,64,65]. The influence of AD and its associated symptoms focused on activities of daily living [59-61,63-68], mental health [59,64-68] and emotional functioning [59-61,63-65,67], relationships [61,63-65,68], independence [59-62,64,68], social functioning [59,61,63-65], role functioning [63,68] and self image [59,61,63,68]. Table 3 provides example quotes to illustrate these concepts.

**Table 3:** Example quotes for AD concepts

Concept	Sub concept	Example quotes
Symptoms	Memory loss	I can look up somebody's name, go to the phone book, once I've got the number, I've forgotten whose name I'm looking for [61]
	
	Loss of thought	This is the worst part when I lose my train of thoughts and you stand there like an idiot! It gets a little embarrassing[61]

Emotional Functioning	Anger	The worst thing is my short term memory, which irritates me so much. I get angry with myself. [65]
	
	Fear	Well, that part is a little frightening when all of a sudden you find yourself, you know, what do I do? Like where am I? or what[61]

Mental Health	Anxiety	It's kind of scary to me...and I'll hate going out or anything. [61]

Relationships	Partner	She wants to help and sometimes she overhelps and I have to say, you know, just leave me, and she gets a bit cross... [59]
	
	Friends	No...my intimate friends possibly, but I don't talk that much about it...this is my concern, and I will sort it out because it afflicts me, me and my family. But our neighbours know[65]
	
	Family	My family members' relationships with me changed as soon as they found out I was 'no longer competent.' The things that I say seem to be a lot more subject to question than they used to be. It's as if I cant possibly know anything anymore[68]

Social Impact	Socialising	Well, all of a sudden I felt a wave of terror wash over me. We went in and it turned out to be a surprise party. Well, I didn't recognize anyone...it was then that I got overcome and passed out. It was the worst experience I can tell you. [63].
	
	Conversation	I'm ducking out of conversations more. [64]
	
	Withdrawal	Yeah, another bad thing that I find now, that I don't want to speak to anybody in here (meaning the housing complex). Because I can't talk to them soon as they talk...I know everybody. But their names are all gone. [61]

Physical functioning	Physical well being	I'm so happy that I'm physically well. So...so I make sure I'm out and about a lot. [65]

Activities of Daily living	Activities	As for driving the car, I used to like it...but now I have to get in a car with someone else and tell them where I want to go[68]
		
		I can look up somebody's name, go to the phone book, once I've got the number, I've forgotten whose name I'm looking for...[61]
	
	Self care	I can't do anything myself. Even buttons and things like that...I just...they do it for me...I hate these things. I get the temper. [64]
	
	Hobbies	I think it started with the sewing. People would ask me to sew something for them and I'd forget all about it [63].

Self Image	Self concept	It's devastating, and it takes away your sense of self. And I find it very hard to deal with [63]

Independence	Loss of independence	Your neighbours will stop and talk to you, just for a minute. Then they'll say, 'well I'll walk around with you.' And I wish I'd never told them I have it because it took away my freedom. [68]

Role Functioning	Work	I used to teach classes. I used to edit a journal. I used to do all kinds of things I'm not doing now[68]

A review of the concepts of relevance to both patients with type 2 diabetes and AD indicate that there are a number of concepts that the two disease areas have in common such as activities of daily living, role functioning, emotional functioning, mental health, relationships, social functioning, and self image. However there are concepts that are relevant to type 2 diabetes such as sexual functioning that were not highlighted as relevant to AD patients, conversely patients with AD were greatly impacted by the loss of their independence where type 2 diabetes patients did not mention this. Finally the key symptoms of each disease were also quite different (fatigue in type 2 diabetes vs. memory loss in AD). Thus, this evidence highlights that while some disease areas do have common concepts there are also important concepts that may differ highlighting a potential lack of sensitivity when measuring utilities using a generic instrument.

### Literature Review Part II: Utility Measures

The additional literature search generated 160 abstracts. The titles and abstracts were reviewed and a total of 145 were excluded due to the search terms not being in the title or abstract, due to duplication between databases or the search terms were not the main focus of the article. A total of 15 articles were reviewed in full. Although no disease-specific utilities were identified, the following generic measures were used in both indications: EQ-5D, 15D, SF-6D, and the HUI (versions 2 and 3). A comparison of the content and measurement properties of these measures is described below.

### EQ-5D

The EQ-5D was developed as a generic, HRQoL instrument designed to assess health outcome states across a wide variety of interventions on a common scale [13]. While healthy people were involved in an exercise using a VAS and a TTO to develop the utility scores, no one was interviewed to ascertain which concepts should be included as a comprehensive assessment of HRQoL. The instrument was developed based on a review of existing literature and health status measures at the time [69]. The EQ-5D index is classified according to five dimensions: mobility, self care, usual activities, pain and mood. There is one additional item where health state "today" is compared with general level of health over the past twelve months. There is also a VAS scale where the current health status is rated on a scale from 0 (worst imaginable health state) to 100 (best imaginable health state).

### 15D

The 15D was developed to measure HRQoL and its utility, and to evaluate the efficacy and effectiveness of interventions [11]. It was developed based on a review of existing literature and instruments and also involved health care professional input. Values were derived based on surveys using the general population [69]. It is a generic fifteen item instrument: mobility, vision, hearing, breathing, eating, sleeping, speech, elimination, usual activities, mental function, discomfort and symptoms, depression, distress, vitality and sexual activity.

### SF-6D

The SF-6D was developed as a simplified health state classification from the SF-36/SF-12 [12] which was developed based on literature and instrument review but did not involve patient or general population input in its development. Values however were generated based on input from the UK general population [69]. It is a generic measure designed to assess HRQoL across age, disease and treatment groups. The SF-6D includes 6 domains: physical functioning, role functioning, social functioning, pain, mental health and vitality.

### Health Utilities Index (HUI)

The HUI consists of three systems: HUI Mark 1 (HUI 1), Mark 2 (HUI 2) and Mark 3 (HUI 3) [10]. HUI 2 and HUI 3 are more frequently used and so have been included in this review. The HUI was developed to describe health status, measure within-attribute morbidity and HRQoL, as well as to produce utility scores. The HUI 2 system appears to have been developed based on theory and literature review and a survey of parents; and the general population were involved in the development of valuation scores [69]. The HUI 2 includes seven attributes: sensation, mobility, emotion, cognition, self care, pain and fertility. The HUI 3 includes eight attributes: vision, hearing, speech, ambulation, dexterity, emotion, cognition and pain. Both are generic instruments.

### Mapping PRO Conceptual Models to Utility Instruments

**Figure 2:**
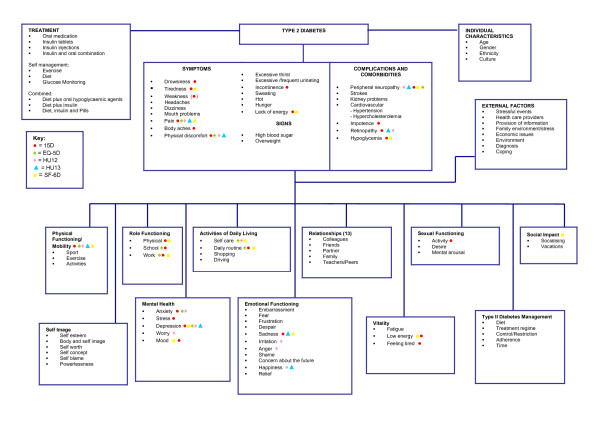
**Impact of Patients with Type 2 Diabetes**.

**Figure 3:**
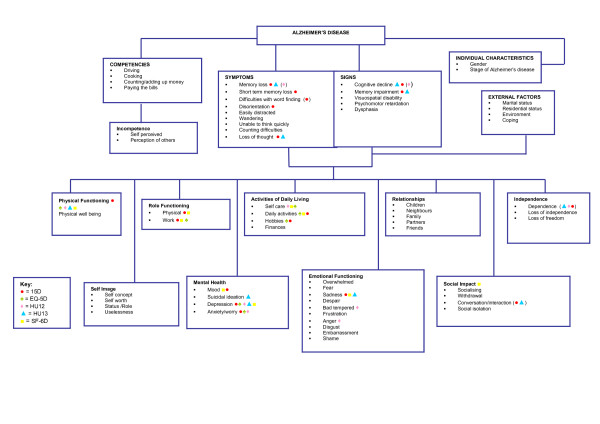
**Impact of Patients with Alzheimer's Disease**.

Based on the qualitative results, Figure 2 and Figure 3 provide proposed conceptual models of type 2 diabetes and AD, respectively. The individual items of the generic utility instruments were then mapped on to the proposed conceptual models. Findings showed that although there were many relevant instrument items, none of the generic utility measures covered all the relevant concepts important to patients with type 2 diabetes or AD. The 15D seemed to be the most comprehensive measure for both type 2 diabetes and AD. The HUI 3 seemed to have the least coverage for the type 2 diabetes conceptual model, and the EQ-5D for the AD conceptual model. The HUI 3 and EQ-5D do not have items relating to relationships, sexual functioning or social impact; all of which emerged as relevant concepts important to patients with type 2 diabetes. Similarly, the EQ-5D does not have items relating to cognitive functioning, social impact or relationships; concepts of relevance to patients with AD.

Further, some of the utility instruments contained items that could not be mapped onto either of the proposed conceptual models. For example, the 'hearing,' 'breathing' and 'sleeping' items from the 15D did not appear to be relevant to either indication which could lead to potential problems such as missing data or lack of sensitivity for these measures when included in a clinical trial.

## Discussion

In general, the findings from our study have demonstrated there was incomplete and varied coverage of relevant concepts across specific disease areas, with disparities varying depending on the disease in question. There were also differences in the concepts covered within the generic utility measures discussed. Both findings point to a potential lack of consistency between measures.

An evaluation of the utility instruments needs to take into account three issues: relevance of concepts to the general population, such as whether a concept specific to a disease would resonate and be relevant to a healthy individual, relevance of concepts to the patient population being assessed and comprehensiveness of the concepts included in the questionnaire from the patient and general population perspective.

To our knowledge, the general population did not rate or rank which concepts were the most important to include in the four utility instruments assessed. All of the concepts included in the utility instruments were based on literature and instrument reviews, rather than using input from patients or the general population. The four instruments also differed in the concepts that were covered. It would be unrealistic to expect that a generic instrument cover each and every concept that is of importance to the general population. However, some concepts that are important to patients may also be important to the population at large. Our results for the diabetes population suggest that tiredness, sexual functioning, social or family impact, and work impact are key concepts that are not covered by many of the utility scales but are likely to be important to the general population. Similarly, cognition and independence are important concepts to AD patients and are likely to be important to the general population. Brazier et al have already demonstrated in some diseases that leaving out concepts considered relevant to patients can result in under representation of relevant health impact in the QALY and over estimation of the cost per QALY [69]. Further testing of these assumptions is warranted in other important diseases such as AD and diabetes.

A detailed calibration of these instruments (as per Brazier et al) in AD and diabetes was beyond the scope of this study. However, the concept mapping illustrates the challenges in representing the diseases optimally and in a way that allows comparison with other diseases. It also raises some philosophical questions about how the content of utility measures should be decided upon. Some instruments may be weighted toward concepts that are of no relevance to the patient population being studied. For example, in our review, patients with AD reported being physically healthy, but the EQ-5D has a preponderance of items that cover physical health (pain, mobility, self-care, usual activities). This leaves only one item (depression) that may be relevant to AD patients and completely omits the key AD concepts of memory and independence. Thus, it is likely that in any economic analysis of current AD drugs that use the EQ-5D as the primary utility measure, the results will only allow assessment of the impact of treatment on depression thereby under representing the full utility of treatment. One could argue that the concepts covered are relevant to the general population and therefore allow for more accurate economic analyses. Yet, it could also be argued that cognition and independence are important concepts to the general population as well as to AD patients, so those concepts perhaps should have been included in a generic utility measure (as indeed they are represented in the 15D and HUI).

It can also be questioned whether it is possible to measure healthy people and people with a relevant disease using the same measure. Qualitative research such as focus groups or in-depth interviews with members of the public that represent typical diseases in the general population should be conducted to ascertain the concepts that are most relevant to include in a HRQL utility measure. This could be considered in the development of reference utility measures. This is in line with recent guidance from reimbursement bodies who suggest that qualitative evidence is also important when valuing utilities. Qualitative evidence provides unique insight into the disease from the patient who has experienced the symptoms and impacts of the disease first hand. This is supported by NICE who state 'Patients and carers are a unique source of expert information about the personal impact of a disease and its treatment' [3].

These findings are consistent with previous research. For example, one study compared three generic utility based measures (EQ-5D, SF-6D and HUI 3) in age-related macular degeneration (ARMD) [70]. Results indicated that the impact of age-related macular degeneration was not well reflected in the EQ-5D or SF-6D with mean utility values of 0.72 (standard deviation = 0.22) and 0.66 (standard deviation = 0.14) respectively. With regard to the HUI 3, which contains a vision domain, this was considered to better reflect the visual functioning impact; with a mean utility value of 0.34 (standard deviation = 0.28) it had a larger and more significant correlation with tests of visual function than the other preference-based measures.

Other studies also provide evidence to support how the choice of method for utility elicitation may lead to difference in utility assessments. For example; one study compared the SF-6D with the EQ-5D and found that there was agreement between both utility instruments demonstrating evidence of validity. However on closer reflection utility results varied, with EQ-5D scores being significantly higher in healthy populations, and SF-6D scores being significantly higher in disease samples, thus pointing to differences in scaling and a lack of calibration between utility measures [71].

Further, a literature review of studies in a variety of disease areas (including but not limited to patients with hearing impairment, spine disorders, rheumatic diseases) that compared preference based systems found that the EQ-5D showed larger change scores and more favorable cost-effectiveness ratios compared to the HUI 2 and HUI 3 [72] and the SF-6D.

The results from these studies and supporting evidence are pertinent given that generic utility measures are documented as the preferred option in reimbursement guidance documents [3-6] and that they are used by national, regional and local key stakeholders in Europe to facilitate decision making regarding allocation of resources across competing interventions. Such findings point towards a need to take in to greater account the patient perspective and relevant concepts as part of the input to decision-making.

Our study has a number of possible limitations that deserve comment. This study was based on published literature, most of which the purpose was not to assess impact on HRQoL. In addition, the literature search was restricted to English language studies and MeSH terms were used when available. MeSH terms were not available for all search terms. Therefore valuable information could have been missed and there may still remain some concepts of importance.

The proposed conceptual models of impact were developed based on existing qualitative research that had been carried out and was from the patient perspective. Consequently, they have not been supplemented with patient interviews to confirm that the proposed areas of impact are indeed important and relevant to patients and put in the context of a specific treatment of study. Having said that, the volume of available information was encouraging as a resource. Additionally, we have limited this study to two indications and the relevant concepts may be very different for others. Further research could explore the relevance of utility instruments in other indications and in the general population.

Also the very nature of AD means that less patient reported information is available, and many studies rely on caregiver reports. The patient reported information that is available is primarily from mild to moderate AD patients and so the proposed models may be reflective of this. Thus the proposed model may be a reflection of the earlier stages of the condition and not the disease in its entirety.

There were other disease associated factors which may influence patients' experiences with their disease. Some studies commented on the added impact of being diagnosed with and the management of diabetes if in an ethnic minority. Diabetes management itself emerged as a seemingly relevant associated factor. Coping strategies appeared to weigh heavily in AD and these included very practical adaptive techniques like using aide memoirs and writing things down, and the use of humour and laughing to cope with the emotional strain associated with the daily hassles of forgetting. Other external factors included age, gender, culture and environment. These other factors may influence the patients' experience with a condition and may have an interactive role in the proposed conceptual models that have been developed from the literature; however such factors would also be controlled for, and taken into account in a clinical trial setting.

A further aspect of the development of future utility instruments is that the dominance of the QALY in the use of HTA and economic evaluation could be reduced by the use of alternative instruments or methods of measurement. Agencies such as the Institute for Quality and Efficiency in Health Care (IQWiG) in Germany and discussions at recent HTA conferences have been considering the use of alternative methods for decision making. Therefore if the QALY is to retain its place as a key method for the use of cost-effectiveness for decision making across countries generic measures need to be constantly improved. Including the patient perspective in development of future instruments and the further development of existing instruments could be a first step aiding the continual improvement of such instruments.

## Conclusions

Despite the limitations of this study, the findings illustrate the potential value of considering qualitative information in the interpretation of cost utility estimates and the need to include more qualitative research in the design of not only the next wave of utility instruments, but also in the further development of existing instruments such as the EQ-5D.

Content of the utility measures has a significant impact on the treatment effects that can be observed and currently in choosing a generic utility measure there appears to be a tradeoff of content in order to achieve consistency. The amount of patient relevant information missing from the chosen generic measures varies from one indication to another, which provides, and could continue to provide a source of inconsistency between the measures. Likewise, the variability in concepts across the generic measures suggests lack of consistency even for general population research, suggesting that the potential impact and importance of content is presently under represented. With current reporting standards, we cannot know the size of this tradeoff between content and consistency and its contribution to the variance in utility findings. This issue would remain if the proposed solution of a "reference method" [21] for all economic evaluations was realised. Thus we propose that a content map of the chosen utility measure with patient-reported domains be provided as standard reporting in cost utility measurement in order to improve the transparency of the trade-offs in relation to patient relevance and consistency.

Patient's perspectives using qualitative methods should be adopted and feature in the decision-making methods used by reimbursement bodies so that there are clearly defined criteria. This movement has the attraction of reducing the scope for controversial decisions based on utility estimates. It could also serve as a bridge to the methods endorsed by regulatory authorities such as the Food and Drug Administration (FDA) and European Medicines Agency (EMA) [16,17] and allow for better continuity of knowledge about treatment effects.

## Abbreviations

AD: Alzheimer's disease; ARMD: Age-related macular degeneration; EM: European Medicines Agency; EQ 5D: EuroQol-5 Dimensions; FDA: Food and Drug Administration; HRQoL: Health-related quality of life; HUI 1: Health Utilities Index Mark 1; HUI 2: Health Utilities Index Mark 2; HUI 3: Health Utilities Index Mark 3; MOS SF-6D: Medical Outcomes Study Health Survey; NICE: National Institute for Health and Clinical Excellence; QALY: Quality-Adjusted Life Year; SMC: Scottish Medicine Consortium; 15D: 15-dimensional health related quality of life measure

## Competing interests

Please note that the data, models and methodology used in the research are not proprietary. With regard to corporate-sponsored research, Pfizer has commissioned Mapi Values, a health outcomes agency, to carry out research on their behalf. The contact of the sponsoring organisation is Clare McGrath.

Diana Rofail works as an Associate Research Director in the Questionnaire and Validation unit of Mapi Values. She has acted as an advisor for various pharmaceutical companies regarding their clinical trials and patient reported outcomes. Diana Rofail does not have ownerships intents (including stock options) in a start up company, the stock of which in not publicly traded or in a publicly traded company. Furthermore, she is not a member of an advisory board or board of directors.

Linda Abetz works as a Director of the Questionnaire and Validation unit of Mapi Values. She has acted as an advisor for various pharmaceutical companies regarding their clinical trials and patient reported outcomes. Linda Abetz does not have ownerships intents (including stock options) in a start up company, the stock of which in not publicly traded or in a publicly traded company. Furthermore, she is not a member of an advisory board or board of directors.

Elizabeth Gargon worked as a Research Associate at Mapi Values. She provided support to Diana Rofail on this project in terms of the presentation and interpretation of results. Elizabeth Gargon does not have ownerships intents (including stock options) in a start up company, the stock of which in not publicly traded or in a publicly traded company. Furthermore, she is not a member of an advisory board or board of directors.

Clare McGrath is Senior Director of HTA Policy at Pfizer and owns stocks and/or stock options. She has had oversight of the HE/OR contributions to licensing and HTA submissions for the past decade and previously specialized in outcomes assessment and clinical studies.

Mapi Values and Pfizer would like to and intend to publish the data irrespective of the review process outcome/comments.

## Authors' contributions

CM conceived of the study, participated in its design and was involved in developing and critically revising the manuscript. DR participated in the design of the study and coordination, analysis and interpretation of the literature review, and developed the manuscript. EG helped to conduct the literature review, participated in the analysis and presentation of results and helped draft the manuscript. LA made contributions to design and interpretation and was involved in the revision of the manuscript. All authors read and approved the final manuscript.
